# Effects of Social Media Use on Psychological Well-Being: A Mediated Model

**DOI:** 10.3389/fpsyg.2021.678766

**Published:** 2021-06-21

**Authors:** Dragana Ostic, Sikandar Ali Qalati, Belem Barbosa, Syed Mir Muhammad Shah, Esthela Galvan Vela, Ahmed Muhammad Herzallah, Feng Liu

**Affiliations:** ^1^School of Finance and Economics, Jiangsu University, Zhenjiang, China; ^2^Research Unit of Governance, Competitiveness, and Public Policies (GOVCOPP), Center for Economics and Finance (cef.up), School of Economics and Management, University of Porto, Porto, Portugal; ^3^Department of Business Administration, Sukkur Institute of Business Administration (IBA) University, Sukkur, Pakistan; ^4^CETYS Universidad, Tijuana, Mexico; ^5^Department of Business Administration, Al-Quds University, Jerusalem, Israel; ^6^Business School, Shandong University, Weihai, China

**Keywords:** smartphone addiction, social isolation, bonding social capital, bridging social capital, phubbing, social media use

## Abstract

The growth in social media use has given rise to concerns about the impacts it may have on users' psychological well-being. This paper's main objective is to shed light on the effect of social media use on psychological well-being. Building on contributions from various fields in the literature, it provides a more comprehensive study of the phenomenon by considering a set of mediators, including social capital types (i.e., bonding social capital and bridging social capital), social isolation, and smartphone addiction. The paper includes a quantitative study of 940 social media users from Mexico, using structural equation modeling (SEM) to test the proposed hypotheses. The findings point to an overall positive indirect impact of social media usage on psychological well-being, mainly due to the positive effect of bonding and bridging social capital. The empirical model's explanatory power is 45.1%. This paper provides empirical evidence and robust statistical analysis that demonstrates both positive and negative effects coexist, helping to reconcile the inconsistencies found so far in the literature.

## Introduction

The use of social media has grown substantially in recent years (Leong et al., 2019; Kemp, 2020). Social media refers to “the websites and online tools that facilitate interactions between users by providing them opportunities to share information, opinions, and interest” (Swar and Hameed, 2017, p. 141). Individuals use social media for many reasons, including entertainment, communication, and searching for information. Notably, adolescents and young adults are spending an increasing amount of time on online networking sites, e-games, texting, and other social media (Twenge and Campbell, 2019). In fact, some authors (e.g., Dhir et al., 2018; Tateno et al., 2019) have suggested that social media has altered the forms of group interaction and its users' individual and collective behavior around the world.

Consequently, there are increased concerns regarding the possible negative impacts associated with social media usage addiction (Swar and Hameed, 2017; Kircaburun et al., 2020), particularly on psychological well-being (Chotpitayasunondh and Douglas, 2016; Jiao et al., 2017; Choi and Noh, 2019; Chatterjee, 2020). Smartphones sometimes distract their users from relationships and social interaction (Chotpitayasunondh and Douglas, 2016; Li et al., 2020a), and several authors have stressed that the excessive use of social media may lead to smartphone addiction (Swar and Hameed, 2017; Leong et al., 2019), primarily because of the fear of missing out (Reer et al., 2019; Roberts and David, 2020). Social media usage has been associated with anxiety, loneliness, and depression (Dhir et al., 2018; Reer et al., 2019), social isolation (Van Den Eijnden et al., 2016; Whaite et al., 2018), and “phubbing,” which refers to the extent to which an individual uses, or is distracted by, their smartphone during face-to-face communication with others (Chotpitayasunondh and Douglas, 2016; Jiao et al., 2017; Choi and Noh, 2019; Chatterjee, 2020).

However, social media use also contributes to building a sense of connectedness with relevant others (Twenge and Campbell, 2019), which may reduce social isolation. Indeed, social media provides several ways to interact both with close ties, such as family, friends, and relatives, and weak ties, including coworkers, acquaintances, and strangers (Chen and Li, 2017), and plays a key role among people of all ages as they exploit their sense of belonging in different communities (Roberts and David, 2020). Consequently, despite the fears regarding the possible negative impacts of social media usage on well-being, there is also an increasing number of studies highlighting social media as a new communication channel (Twenge and Campbell, 2019; Barbosa et al., 2020), stressing that it can play a crucial role in developing one's presence, identity, and reputation, thus facilitating social interaction, forming and maintaining relationships, and sharing ideas (Carlson et al., 2016), which consequently may be significantly correlated to social support (Chen and Li, 2017; Holliman et al., 2021). Interestingly, recent studies (e.g., David et al., 2018; Bano et al., 2019; Barbosa et al., 2020) have suggested that the impact of smartphone usage on psychological well-being depends on the time spent on each type of application and the activities that users engage in.

Hence, the literature provides contradictory cues regarding the impacts of social media on users' well-being, highlighting both the possible negative impacts and the social enhancement it can potentially provide. In line with views on the need to further investigate social media usage (Karikari et al., 2017), particularly regarding its societal implications (Jiao et al., 2017), this paper argues that there is an urgent need to further understand the impact of the time spent on social media on users' psychological well-being, namely by considering other variables that mediate and further explain this effect.

One of the relevant perspectives worth considering is that provided by social capital theory, which is adopted in this paper. Social capital theory has previously been used to study how social media usage affects psychological well-being (e.g., Bano et al., 2019). However, extant literature has so far presented only partial models of associations that, although statistically acceptable and contributing to the understanding of the scope of social networks, do not provide as comprehensive a vision of the phenomenon as that proposed within this paper. Furthermore, the contradictory views, suggesting both negative (e.g., Chotpitayasunondh and Douglas, 2016; Van Den Eijnden et al., 2016; Jiao et al., 2017; Whaite et al., 2018; Choi and Noh, 2019; Chatterjee, 2020) and positive impacts (Carlson et al., 2016; Chen and Li, 2017; Twenge and Campbell, 2019) of social media on psychological well-being, have not been adequately explored.

Given this research gap, this paper's main objective is to shed light on the effect of social media use on psychological well-being. As explained in detail in the next section, this paper explores the mediating effect of bonding and bridging social capital. To provide a broad view of the phenomenon, it also considers several variables highlighted in the literature as affecting the relationship between social media usage and psychological well-being, namely smartphone addiction, social isolation, and phubbing. The paper utilizes a quantitative study conducted in Mexico, comprising 940 social media users, and uses structural equation modeling (SEM) to test a set of research hypotheses.

This article provides several contributions. First, it adds to existing literature regarding the effect of social media use on psychological well-being and explores the contradictory indications provided by different approaches. Second, it proposes a conceptual model that integrates complementary perspectives on the direct and indirect effects of social media use. Third, it offers empirical evidence and robust statistical analysis that demonstrates that both positive and negative effects coexist, helping resolve the inconsistencies found so far in the literature. Finally, this paper provides insights on how to help reduce the potential negative effects of social media use, as it demonstrates that, through bridging and bonding social capital, social media usage positively impacts psychological well-being. Overall, the article offers valuable insights for academics, practitioners, and society in general.

The remainder of this paper is organized as follows. Section Literature Review presents a literature review focusing on the factors that explain the impact of social media usage on psychological well-being. Based on the literature review, a set of hypotheses are defined, resulting in the proposed conceptual model, which includes both the direct and indirect effects of social media usage on psychological well-being. Section Research Methodology explains the methodological procedures of the research, followed by the presentation and discussion of the study's results in section Results. Section Discussion is dedicated to the conclusions and includes implications, limitations, and suggestions for future research.

## Literature Review

Putnam (1995, p. 664–665) defined social capital as “features of social life – networks, norms, and trust – that enable participants to act together more effectively to pursue shared objectives.” Li and Chen (2014, p. 117) further explained that social capital encompasses “resources embedded in one's social network, which can be assessed and used for instrumental or expressive returns such as mutual support, reciprocity, and cooperation.”

Putnam (1995, 2000) conceptualized social capital as comprising two dimensions, bridging and bonding, considering the different norms and networks in which they occur. Bridging social capital refers to the inclusive nature of social interaction and occurs when individuals from different origins establish connections through social networks. Hence, bridging social capital is typically provided by heterogeneous weak ties (Li and Chen, 2014). This dimension widens individual social horizons and perspectives and provides extended access to resources and information. Bonding social capital refers to the social and emotional support each individual receives from his or her social networks, particularly from close ties (e.g., family and friends).

Overall, social capital is expected to be positively associated with psychological well-being (Bano et al., 2019). Indeed, Williams (2006) stressed that interaction generates affective connections, resulting in positive impacts, such as emotional support. The following sub-sections use the lens of social capital theory to explore further the relationship between the use of social media and psychological well-being.

### Social Media Use, Social Capital, and Psychological Well-Being

The effects of social media usage on social capital have gained increasing scholarly attention, and recent studies have highlighted a positive relationship between social media use and social capital (Brown and Michinov, 2019; Tefertiller et al., 2020). Li and Chen (2014) hypothesized that the intensity of Facebook use by Chinese international students in the United States was positively related to social capital forms. A longitudinal survey based on the quota sampling approach illustrated the positive effects of social media use on the two social capital dimensions (Chen and Li, 2017). Abbas and Mesch (2018) argued that, as Facebook usage increases, it will also increase users' social capital. Karikari et al. (2017) also found positive effects of social media use on social capital. Similarly, Pang (2018) studied Chinese students residing in Germany and found positive effects of social networking sites' use on social capital, which, in turn, was positively associated with psychological well-being. Bano et al. (2019) analyzed the 266 students' data and found positive effects of WhatsApp use on social capital forms and the positive effect of social capital on psychological well-being, emphasizing the role of social integration in mediating this positive effect.

Kim and Kim (2017) stressed the importance of having a heterogeneous network of contacts, which ultimately enhances the potential social capital. Overall, the manifest and social relations between people from close social circles (bonding social capital) and from distant social circles (bridging social capital) are strengthened when they promote communication, social support, and the sharing of interests, knowledge, and skills, which are shared with other members. This is linked to positive effects on interactions, such as acceptance, trust, and reciprocity, which are related to the individuals' health and psychological well-being (Bekalu et al., 2019), including when social media helps to maintain social capital between social circles that exist outside of virtual communities (Ellison et al., 2007).

Grounded on the above literature, this study proposes the following hypotheses:

H1a: Social media use is positively associated with bonding social capital.
H1b: Bonding social capital is positively associated with psychological well-being.
H2a: Social media use is positively associated with bridging social capital.
H2b: Bridging social capital is positively associated with psychological well-being.

### Social Media Use, Social Isolation, and Psychological Well-Being

Social isolation is defined as “a deficit of personal relationships or being excluded from social networks” (Choi and Noh, 2019, p. 4). The state that occurs when an individual lacks true engagement with others, a sense of social belonging, and a satisfying relationship is related to increased mortality and morbidity (Primack et al., 2017). Those who experience social isolation are deprived of social relationships and lack contact with others or involvement in social activities (Schinka et al., 2012). Social media usage has been associated with anxiety, loneliness, and depression (Dhir et al., 2018; Reer et al., 2019), and social isolation (Van Den Eijnden et al., 2016; Whaite et al., 2018). However, some recent studies have argued that social media use decreases social isolation (Primack et al., 2017; Meshi et al., 2020). Indeed, the increased use of social media platforms such as Facebook, WhatsApp, Instagram, and Twitter, among others, may provide opportunities for decreasing social isolation. For instance, the improved interpersonal connectivity achieved via videos and images on social media helps users evidence intimacy, attenuating social isolation (Whaite et al., 2018).

Chappell and Badger (1989) stated that social isolation leads to decreased psychological well-being, while Choi and Noh (2019) concluded that greater social isolation is linked to increased suicide risk. Schinka et al. (2012) further argued that, when individuals experience social isolation from siblings, friends, family, or society, their psychological well-being tends to decrease. Thus, based on the literature cited above, this study proposes the following hypotheses:

H3a: Social media use is significantly associated with social isolation.
H3b: Social isolation is negatively associated with psychological well-being.

### Social Media Use, Smartphone Addiction, Phubbing, and Psychological Well-Being

Smartphone addiction refers to “an individuals' excessive use of a smartphone and its negative effects on his/her life as a result of his/her inability to control his behavior” (Gökçearslan et al., 2018, p. 48). Regardless of its form, smartphone addiction results in social, medical, and psychological harm to people by limiting their ability to make their own choices (Chotpitayasunondh and Douglas, 2016). The rapid advancement of information and communication technologies has led to the concept of social media, e-games, and also to smartphone addiction (Chatterjee, 2020). The excessive use of smartphones for social media use, entertainment (watching videos, listening to music), and playing e-games is more common amongst people addicted to smartphones (Jeong et al., 2016). In fact, previous studies have evidenced the relationship between social use and smartphone addiction (Salehan and Negahban, 2013; Jeong et al., 2016; Swar and Hameed, 2017). In line with this, the following hypotheses are proposed:

H4a: Social media use is positively associated with smartphone addiction.
H4b: Smartphone addiction is negatively associated with psychological well-being.

While smartphones are bringing individuals closer, they are also, to some extent, pulling people apart (Tonacci et al., 2019). For instance, they can lead to individuals ignoring others with whom they have close ties or physical interactions; this situation normally occurs due to extreme smartphone use (i.e., at the dinner table, in meetings, at get-togethers and parties, and in other daily activities). This act of ignoring others is called phubbing and is considered a common phenomenon in communication activities (Guazzini et al., 2019; Chatterjee, 2020). Phubbing is also referred to as an act of snubbing others (Chatterjee, 2020). This term was initially used in May 2012 by an Australian advertising agency to describe the “growing phenomenon of individuals ignoring their families and friends who were called phubbee (a person who is a recipients of phubbing behavior) victim of phubber (a person who start phubbing her or his companion)” (Chotpitayasunondh and Douglas, 2018). Smartphone addiction has been found to be a determinant of phubbing (Kim et al., 2018). Other recent studies have also evidenced the association between smartphones and phubbing (Chotpitayasunondh and Douglas, 2016; Guazzini et al., 2019; Tonacci et al., 2019; Chatterjee, 2020). Vallespín et al. (2017) argued that phubbing behavior has a negative influence on psychological well-being and satisfaction. Furthermore, smartphone addiction is considered responsible for the development of new technologies. It may also negatively influence individual's psychological proximity (Chatterjee, 2020). Therefore, based on the above discussion and calls for the association between phubbing and psychological well-being to be further explored, this study proposes the following hypotheses:

H5: Smartphone addiction is positively associated with phubbing.
H6: Phubbing is negatively associated with psychological well-being.

### Indirect Relationship Between Social Media Use and Psychological Well-Being

**Figure 1:**
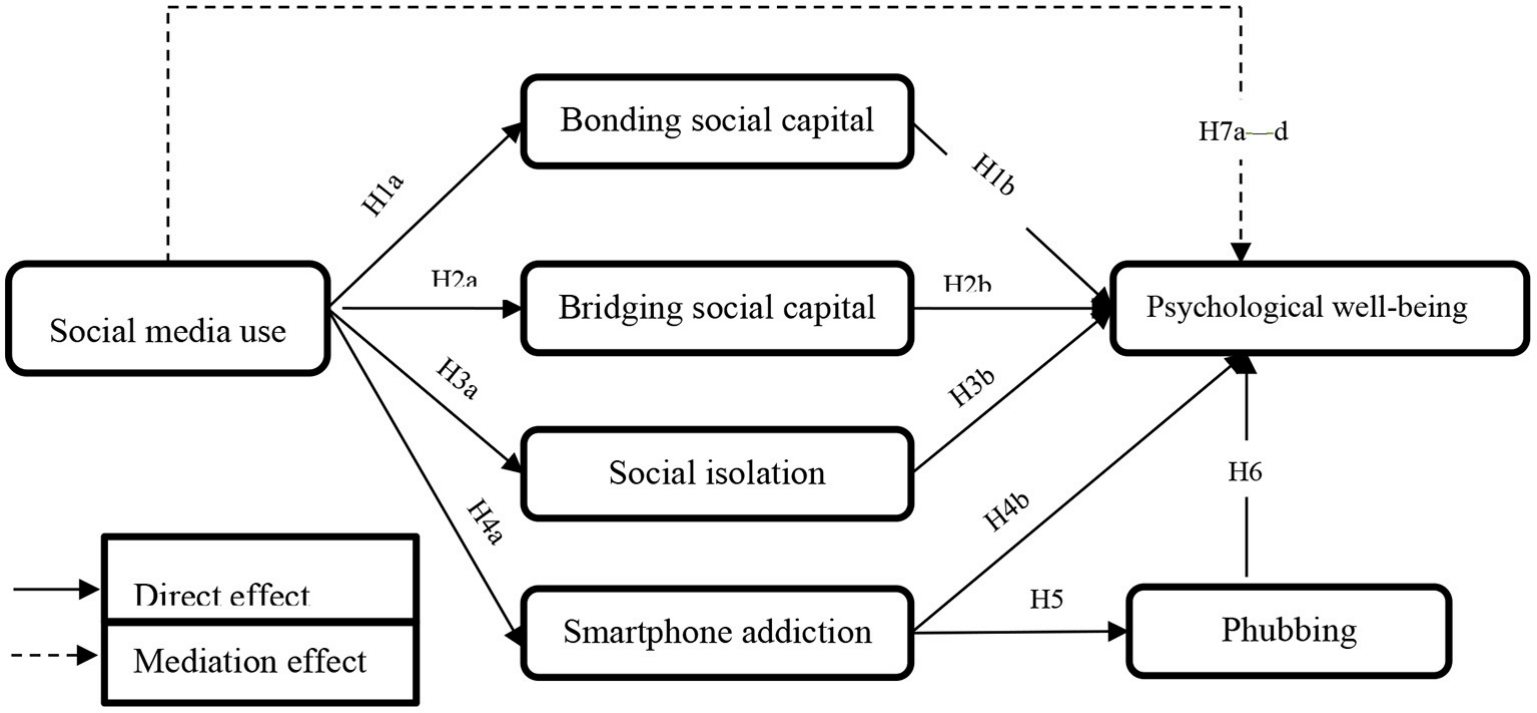
Conceptual model.

Beyond the direct hypotheses proposed above, this study investigates the indirect effects of social media use on psychological well-being mediated by social capital forms, social isolation, and phubbing. As described above, most prior studies have focused on the direct influence of social media use on social capital forms, social isolation, smartphone addiction, and phubbing, as well as the direct impact of social capital forms, social isolation, smartphone addiction, and phubbing on psychological well-being. Very few studies, however, have focused on and evidenced the mediating role of social capital forms, social isolation, smartphone addiction, and phubbing derived from social media use in improving psychological well-being (Chen and Li, 2017; Pang, 2018; Bano et al., 2019; Choi and Noh, 2019). Moreover, little is known about smartphone addiction's mediating role between social media use and psychological well-being. Therefore, this study aims to fill this gap in the existing literature by investigating the mediation of social capital forms, social isolation, and smartphone addiction. Further, examining the mediating influence will contribute to a more comprehensive understanding of social media use on psychological well-being via the mediating associations of smartphone addiction and psychological factors. Therefore, based on the above, we propose the following hypotheses (the conceptual model is presented in Figure 1):

H7: (a) Bonding social capital; (b) bridging social capital; (c) social isolation; and (d) smartphone addiction mediate the relationship between social media use and psychological well-being.

## Research Methodology

### Sample Procedure and Online Survey

This study randomly selected students from universities in Mexico. We chose University students for the following reasons. First, students are considered the most appropriate sample for e-commerce studies, particularly in the social media context (Oghazi et al., 2018; Shi et al., 2018). Second, University students are considered to be frequent users and addicted to smartphones (Mou et al., 2017; Stouthuysen et al., 2018). Third, this study ensured that respondents were experienced, well-educated, and possessed sufficient knowledge of the drawbacks of social media and the extreme use of smartphones. A total sample size of 940 University students was ultimately achieved from the 1,500 students contacted, using a convenience random sampling approach, due both to the COVID-19 pandemic and budget and time constraints. Additionally, in order to test the model, a quantitative empirical study was conducted, using an online survey method to collect data. This study used a web-based survey distributed via social media platforms for two reasons: the COVID-19 pandemic; and to reach a large number of respondents (Qalati et al., 2021). Furthermore, online surveys are considered a powerful and authenticated tool for new research (Fan et al., 2021), while also representing a fast, simple, and less costly approach to collecting data (Dutot and Bergeron, 2016).

### Data Collection Procedures and Respondent's Information

Data were collected by disseminating a link to the survey by e-mail and social network sites. Before presenting the closed-ended questionnaire, respondents were assured that their participation would remain voluntary, confidential, and anonymous. Data collection occurred from July 2020 to December 2020 (during the pandemic). It should be noted that, because data were collected during the pandemic, this may have had an influence on the results of the study. The reason for choosing a six-month lag time was to mitigate common method bias (CMB) (Li et al., 2020b). In the present study, 1,500 students were contacted via University e-mail and social applications (Facebook, WhatsApp, and Instagram). We sent a reminder every month for 6 months (a total of six reminders), resulting in 940 valid responses. Thus, 940 (62.6% response rate) responses were used for hypotheses testing.

Table 1 reveals that, of the 940 participants, three-quarters were female (76.4%, *n* = 719) and nearly one-quarter (23.6%, *n* = 221) were male. Nearly half of the participants (48.8%, *n* = 459) were aged between 26 and 35 years, followed by 36 to 35 years (21.9%, *n* = 206), <26 (20.3%, *n* = 191), and over 45 (8.9%, *n* = 84). Approximately two-thirds (65%, *n* = 611) had a bachelor's degree or above, while one-third had up to 12 years of education. Regarding the daily frequency of using the Internet, nearly half (48.6%, *n* = 457) of the respondents reported between 5 and 8 h a day, and over one-quarter (27.2%) 9–12 h a day. Regarding the social media platforms used, over 38.5 and 39.6% reported Facebook and WhatsApp, respectively. Of the 940 respondents, only 22.1% reported Instagram (12.8%) and Twitter (9.2%). It should be noted, however, that the sample is predominantly female and well-educated.

**Table 1:** Respondents' characteristics.

**Respondents' characteristics**		**Frequency**	**Percent**
**GENDER**			
	Female	719	76.489
	Male	221	23.510
**AGE (YEARS)**			
	<26	191	20.319
	26–35	459	48.829
	36–45	206	21.914
	> 45	84	8.936
**EDUCATION LEVEL**			
	Up to 12 years of education	329	35.000
	Bachelor's degree or above	611	65.000
**FREQUENCY OF USING INTERNET (h)**			
	<4	118	12.553
	5–8	457	48.617
	9–12	256	27.234
	> 12	109	11.595
**SOCIAL MEDIA PLATFORM**			
	Facebook	362	38.510
	WhatsApp	370	39.361
	Instagram	121	12.872
	Twitter	87	9.255

### Measurement Items

The study used five-point Likert scales (1 = “strongly disagree;” 5 = “strongly agree”) to record responses.

#### Social Media Use

Social media use was assessed using four items adapted from Karikari et al. (2017). Sample items include “Social media is part of my everyday activity,” “Social media has become part of my daily life,” “I would be sorry if social media shut down,” and “I feel out of touch, when I have not logged onto social media for a while.” The adapted items had robust reliability and validity (CA = 783, CR = 0.857, AVE = 0.600).

#### Social Capital

Social capital was measured using a total of eight items, representing bonding social capital (four items) and bridging social capital (four items) adapted from Chan (2015). Sample construct items include: bonging social capital (“I am willing to spend time to support general community activities,” “I interact with people who are quite different from me”) and bridging social capital (“My social media community is a good place to be,” “Interacting with people on social media makes me want to try new things”). The adapted items had robust reliability and validity [bonding social capital (CA = 0.785, CR = 0.861, AVE = 0.608) and bridging social capital (CA = 0.834, CR = 0.883, AVE = 0.601)].

#### Social Isolation

Social isolation was assessed using three items from Choi and Noh (2019). Sample items include “I do not have anyone to play with,” “I feel alone from people,” and “I have no one I can trust.” This adapted scale had substantial reliability and validity (CA = 0.890, CR = 0.928, AVE = 0.811).

#### Smartphone Addiction

Smartphone addiction was assessed using five items taken from Salehan and Negahban (2013). Sample items include “I am always preoccupied with my mobile,” “Using my mobile phone keeps me relaxed,” and “I am not able to control myself from frequent use of mobile phones.” Again, these adapted items showed substantial reliability and validity (CA = 903, CR = 0.928, AVE = 0.809).

#### Phubbing

Phubbing was assessed using four items from Chotpitayasunondh and Douglas (2018). Sample items include: “I have conflicts with others because I am using my phone” and “I would rather pay attention to my phone than talk to others.” This construct also demonstrated significant reliability and validity (CA = 770, CR = 0.894, AVE = 0.809).

#### Psychological Well-Being

Psychological well-being was assessed using five items from Jiao et al. (2017). Sample items include “I lead a purposeful and meaningful life with the help of others,” “My social relationships are supportive and rewarding in social media,” and “I am engaged and interested in my daily on social media.” This study evidenced that this adapted scale had substantial reliability and validity (CA = 0.886, CR = 0.917, AVE = 0.688).

### Data Analysis

Based on the complexity of the association between the proposed construct and the widespread use and acceptance of SmartPLS 3.0 in several fields (Hair et al., 2019), we utilized SEM, using SmartPLS 3.0, to examine the relationships between constructs. Structural equation modeling is a multivariate statistical analysis technique that is used to investigate relationships. Further, it is a combination of factor and multivariate regression analysis, and is employed to explore the relationship between observed and latent constructs.

SmartPLS 3.0 “is a more comprehensive software program with an intuitive graphical user interface to run partial least square SEM analysis, certainly has had a massive impact” (Sarstedt and Cheah, 2019). According to Ringle et al. (2015), this commercial software offers a wide range of algorithmic and modeling options, improved usability, and user-friendly and professional support. Furthermore, Sarstedt and Cheah (2019) suggested that structural equation models enable the specification of complex interrelationships between observed and latent constructs. Hair et al. (2019) argued that, in recent years, the number of articles published using partial least squares SEM has increased significantly in contrast to covariance-based SEM. In addition, partial least squares SEM using SmartPLS is more appealing for several scholars as it enables them to predict more complex models with several variables, indicator constructs, and structural paths, instead of imposing distributional assumptions on the data (Hair et al., 2019). Therefore, this study utilized the partial least squares SEM approach using SmartPLS 3.0.

## Results

### Common Method Bias (CMB) Test

This study used the Kaiser–Meyer–Olkin (KMO) test to measure the sampling adequacy and ensure data suitability. The KMO test result was 0.874, which is greater than an acceptable threshold of 0.50 (Ali Qalati et al., 2021; Shrestha, 2021), and hence considered suitable for explanatory factor analysis. Moreover, Bartlett's test results demonstrated a significance level of 0.001, which is considered good as it is below the accepted threshold of 0.05.

The term CMB is associated with Campbell and Fiske (1959), who highlighted the importance of CMB and identified that a portion of variance in the research may be due to the methods employed. It occurs when all scales of the study are measured at the same time using a single questionnaire survey (Podsakoff and Organ, 1986); subsequently, estimates of the relationship among the variables might be distorted by the impacts of CMB. It is considered a serious issue that has a potential to “jeopardize” the validity of the study findings (Tehseen et al., 2017). There are several reasons for CMB: (1) it mainly occurs due to response “tendencies that raters can apply uniformity across the measures;” and (2) it also occurs due to similarities in the wording and structure of the survey items that produce similar results (Jordan and Troth, 2019). Harman's single factor test and a full collinearity approach were employed to ensure that the data was free from CMB (Tehseen et al., 2017; Jordan and Troth, 2019; Ali Qalati et al., 2021). Harman's single factor test showed a single factor explained only 22.8% of the total variance, which is far below the 50.0% acceptable threshold (Podsakoff et al., 2003).

Additionally, the variance inflation factor (VIF) was used, which is a measure of the amount of multicollinearity in a set of multiple regression constructs and also considered a way of detecting CMB (Hair et al., 2019). Hair et al. (2019) suggested that the acceptable threshold for the VIF is 3.0; as the computed VIFs for the present study ranged from 1.189 to 1.626, CMB is not a key concern (see Table 2). Bagozzi et al. (1991) suggested a correlation-matrix procedure to detect CMB. Common method bias is evident if correlation among the principle constructs is >0.9 (Tehseen et al., 2020); however, no values >0.9 were found in this study (see section Assessment of Measurement Model). This study used a two-step approach to evaluate the measurement model and the structural model.

**Table 2:** Common method bias (full collinearity VIF).

**Construct**	**Inner VIF**
Social media use	1.391
Bonding social capital	1.626
Bridging social capital	1.560
Social isolation	1.193
Smartphone addiction	1.408
Phubbing	1.189

### Assessment of Measurement Model

Before conducting the SEM analysis, the measurement model was assessed to examine individual item reliability, internal consistency, and convergent and discriminant validity. Table 3 exhibits the values of outer loading used to measure an individual item's reliability (Hair et al., 2012). Hair et al. (2017) proposed that the value for each outer loading should be ≥0.7; following this principle, two items of phubbing (PHUB3—I get irritated if others ask me to get off my phone and talk to them; PHUB4—I use my phone even though I know it irritated others) were removed from the analysis Hair et al. (2019). According to Nunnally (1978), Cronbach's alpha values should exceed 0.7. The threshold values of constructs in this study ranged from 0.77 to 0.903. Regarding internal consistency, Bagozzi and Yi (1988) suggested that composite reliability (CR) should be ≥0.7. The coefficient value for CR in this study was between 0.857 and 0.928. Regarding convergent validity, Fornell and Larcker (1981) suggested that the average variance extracted (AVE) should be ≥0.5. Average variance extracted values in this study were between 0.60 and 0.811. Finally, regarding discriminant validity, according to Fornell and Larcker (1981), the square root of the AVE for each construct should exceed the inter-correlations of the construct with other model constructs. That was the case in this study, as shown in Table 4.

**Table 3:** Study measures, factor loading, and the constructs' reliability and convergent validity.

**Construct**	**Item code**	**Loading**	**CA**	**CR**	**AVE**
Social media use	SMU1—Social media is part of my everyday activity	0.756	0.783	0.857	0.600
	SMU2—Social media has become part of my daily routine	0.758			
	SMU3—I feel out of touch when I have not logged onto social media for a while	0.834			
	SMU4—I would be sorry if social media shut down	0.747			
Bonding social capital	BoSC1—Based on the people I interact with; it is easy for me to hear about the latest news and trends	0.781	0.785	0.861	0.608
	BoSC2—Interacting with people makes me curious about things and places outside of my daily life	0.829			
	BoSC3—I am willing to spend time to support general community activities	0.793			
	BoSC4—I interact with people who are quite different from me	0.710			
Bridging social capital	BrSC1—I am interested in what goes on in my social media community	0.706	0.834	0.883	0.601
	BrSC2—My social media community is a good place to be	0.786			
	BrSC3—Interacting with people on social media makes me want to try new things	0.749			
	BrSC4—Interacting with people on social media makes me feel like part of a larger community	0.831			
Social isolation	SI1—I do not have anyone to play with	0.923	0.890	0.928	0.811
	SI2—I feel alone from people	0.931			
	SI3—I have no one I can trust	0.846			
Smartphone addiction	SPA1—I am always preoccupied with my mobile phone	0.793	0.903	0.928	0.723
	SPA2—Using my mobile phone keeps me relaxed	0.783			
	SPA3—I feel restless or irritable when attempting to cut down mobile phone use	0.904			
	SPA4—I can't stay even for a moment without a mobile phone	0.884			
	SPA5—I am not able to control myself from frequent use of mobile phone	0.879			
Phubbing	PHUB1—I have conflicts with others because I am using my phone	0.933	0.770	0.894	0.809
	PHUB2—I would rather pay attention to my phone and talk to them	0.865			
Psychological well-being	PWB1—I lead a purposeful and meaningful life with the help of social media	0.826	0.886	0.917	0.688
	PWB2—My social relationships are supportive and rewarding in social media	0.793			
	PWB3—I am engaged and interested in my daily activities on social media	0.868			
	PWB4—I actively contributes to the happiness and well-being of others on social media	0.825			
	PWB5—I am optimistic about my future with the help of social media	0.834			

**Table 4:** Discriminant validity and correlation.

**Construct**	**1**	**2**	**3**	**4**	**5**	**6**	**7**
Bonding social capital	**0.779**						
Bridging social capital	0.464	**0.776**					
Phubbing	0.017	0.242	**0.899**				
Psychological well-being	0.414	0.641	0.243	**0.829**			
Smartphone addiction	−0.290	0.121	0.244	−0.019	**0.850**		
Social isolation	−0.098	0.087	0.305	0.005	0.319	**0.901**	
Social media use	0.332	0.440	0.174	0.343	0.224	0.146	**0.775**

*Bold values are the square root of the AVE*.

Hence, by analyzing the results of the measurement model, it can be concluded that the data are adequate for structural equation estimation.

### Assessment of the Structural Model

**Figure 2:**
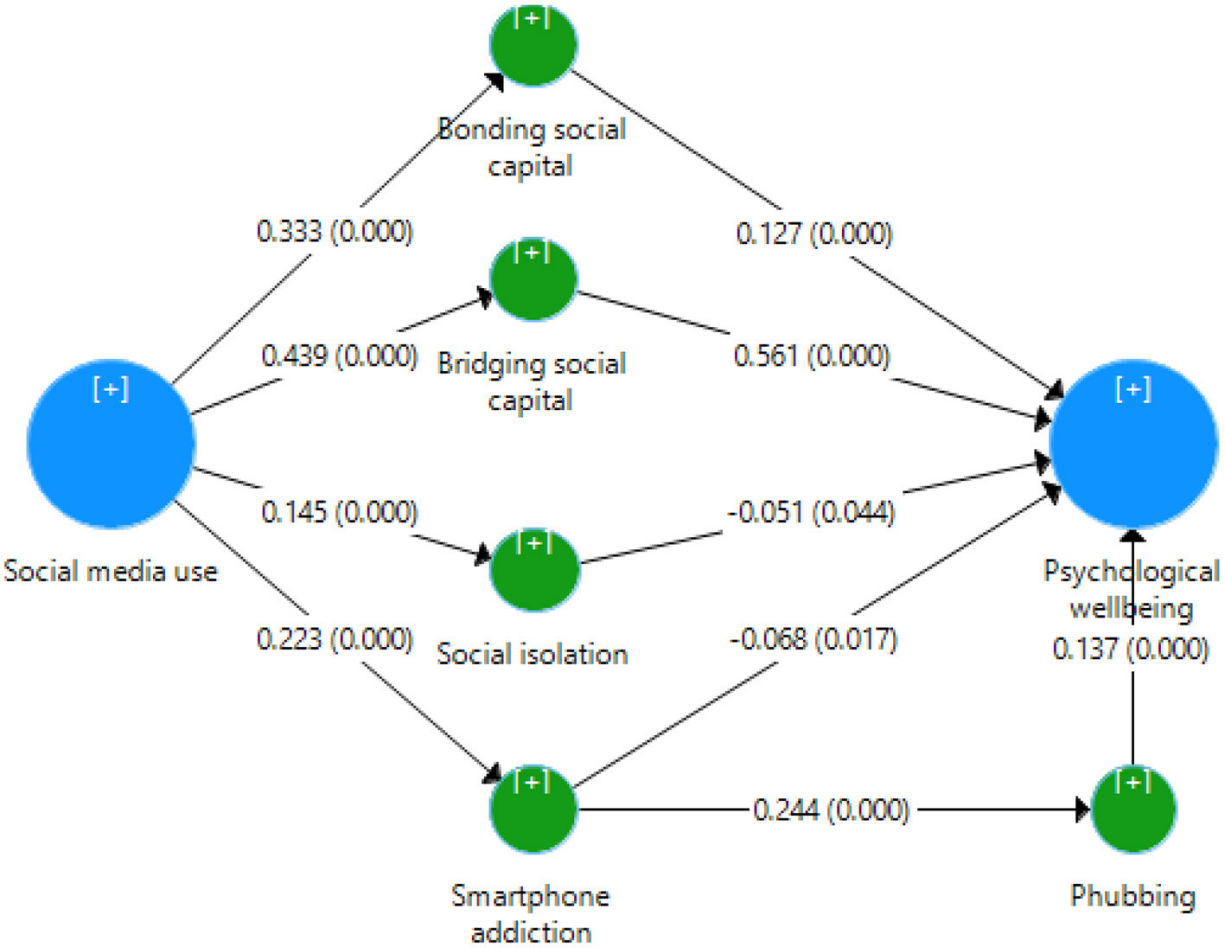
Structural model.

This study used the PLS algorithm and a bootstrapping technique with 5,000 bootstraps as proposed by Hair et al. (2019) to generate the path coefficient values and their level of significance. The coefficient of determination (*R*^2^) is an important measure to assess the structural model and its explanatory power (Henseler et al., 2009; Hair et al., 2019). Table 5 and Figure 2 reveal that the *R*^2^ value in the present study was 0.451 for psychological well-being, which means that 45.1% of changes in psychological well-being occurred due to social media use, social capital forms (i.e., bonding and bridging), social isolation, smartphone addiction, and phubbing. Cohen (1998) proposed that *R*^2^ values of 0.60, 0.33, and 0.19 are considered substantial, moderate, and weak. Following Cohen's (1998) threshold values, this research demonstrates a moderate predicting power for psychological well-being among Mexican respondents (Table 6).

**Table 5:** Summary of path coefficients and hypothesis testing.

**Hypothesis**	**Relationship**	**Path coefficient**	***SD***	***t*-value**	***p*-value**	**Decision**
**DIRECT EFFECT**						
H1a	Social media use → Bonding social capital	0.332	0.032	10.283^*^	0.001	Accepted
H1b	Bonding social capital → Psychological well-being	0.127	0.031	4.077^*^	0.001	Accepted
H2a	Social media use → Bridging social capital	0.439	0.028	15.543^*^	0.001	Accepted
H2b	Bridging social capital → Psychological well-being	0.561	0.027	20.953^*^	0.001	Accepted
H3a	Social media use → Social isolation	0.145	0.029	4.985^*^	0.001	Accepted
H3b	Social isolation → Psychological well-being	−0.051	0.025	2.010^*^	0.044	Accepted
H4a	Social media use → Smartphone addiction	0.223	0.036	6.241^*^	0.001	Accepted
H4b	Smartphone addiction → Psychological well-being	−0.068	0.028	2.387^*^	0.017	Accepted
H5	Smartphone addiction → Phubbing	0.244	0.032	7.555^*^	0.001	Accepted
H6	Phubbing → Psychological well-being	0.137	0.028	4.938^*^	0.001	Accepted
**INDIRECT EFFECT**						
H7a	Social media use → Bonding social capital → Psychological well-being	0.042	0.011	3.740^*^	0.002	Accepted
H7b	Social media use → Bridging social capital → Psychological well-being	0.246	0.021	11.677^*^	0.001	Accepted
H7c	Social media use → Social isolation → Psychological well-being	−0.080	0.004	1.987^*^	0.047	Accepted
H7d	Social media use → Smartphone addiction → Psychological well-being	−0.019	0.008	2.528^*^	0.011	Accepted

* *p-value < 0.05, t-value > 1.96*.

**Table 6:** Strength of the model (Predictive relevance, coefficient of determination, and model fit indices).

	**Effect size**			**Coefficient of determination**	
**Construct**	**SSO**	**SSE**	***Q*^2^ (=1 – SSE/SSO)**	***R*^**2**^**	**Adj. *R*^**2**^**
Psychological well-being	4,700.00	4,543.37	0.29	0.451	0.447

*Goodness of fit → SRMR = 0.063; d_ULS = 1.589; d_G = 0.512; chi-square = 2,910.744*.

Apart from the *R*^2^ measure, the present study also used cross-validated redundancy measures, or effect sizes (*q*^2^), to assess the proposed model and validate the results (Ringle et al., 2012). Hair et al. (2019) suggested that a model exhibiting an effect size *q*^2^ > 0 has predictive relevance (Table 6). This study's results evidenced that it has a 0.15 <0.29 <0.35 (medium) predictive relevance, as 0.02, 0.15, and 0.35 are considered small, medium, and large, respectively (Cohen, 1998). Regarding the goodness-of-fit indices, Hair et al. (2019) suggested the standardized root mean square residual (SRMR) to evaluate the goodness of fit. Standardized root mean square is an absolute measure of fit: a value of zero indicates perfect fit and a value <0.08 is considered good fit (Hair et al., 2019). This study exhibits an adequate model fitness level with an SRMR value of 0.063 (Table 6).

Table 5 reveals that all hypotheses of the study were accepted base on the criterion (*p*-value < 0.05). *H1a* (β = 0.332, *t* = 10.283, *p* = 0.001) was confirmed, with the second most robust positive and significant relationship (between social media use and bonding social capital). In addition, this study evidenced a positive and significant relationship between bonding social capital and psychological well-being (β = 0.127, *t* = 4.077, *p* = 0.001); therefore, *H1b* was accepted. Regarding social media use and bridging social capital, the present study found the most robust positive and significant impact (β = 0.439, *t* = 15.543, *p* = 0.001); therefore, *H2a* was accepted. The study also evidenced a positive and significant association between bridging social capital and psychological well-being (β = 0.561, *t* = 20.953, *p* = 0.001); thus, *H2b* was accepted. The present study evidenced a significant effect of social media use on social isolation (β = 0.145, *t* = 4.985, *p* = 0.001); thus, *H3a* was accepted. In addition, this study accepted *H3b* (β = −0.051, *t* = 2.01, *p* = 0.044). Furthermore, this study evidenced a positive and significant effect of social media use on smartphone addiction (β = 0.223, *t* = 6.241, *p* = 0.001); therefore, *H4a* was accepted. Furthermore, the present study found that smartphone addiction has a negative significant influence on psychological well-being (β = −0.068, *t* = 2.387, *p* = 0.017); therefore, *H4b* was accepted. Regarding the relationship between smartphone addiction and phubbing, this study found a positive and significant effect of smartphone addiction on phubbing (β = 0.244, *t* = 7.555, *p* = 0.001); therefore, *H5* was accepted. Furthermore, the present research evidenced a positive and significant influence of phubbing on psychological well-being (β = 0.137, *t* = 4.938, *p* = 0.001); therefore, *H6* was accepted. Finally, the study provides interesting findings on the indirect effect of social media use on psychological well-being (*t*-value > 1.96 and *p*-value < 0.05); therefore, *H7a–d* were accepted.

Furthermore, to test the mediating analysis, Preacher and Hayes's (2008) approach was used. The key characteristic of an indirect relationship is that it involves a third construct, which plays a mediating role in the relationship between the independent and dependent constructs. Logically, the effect of A (independent construct) on C (the dependent construct) is mediated by B (a third variable). Preacher and Hayes (2008) suggested the following: B is a construct acting as a mediator if A significantly influences B, A significantly accounts for variability in C, B significantly influences C when controlling for A, and the influence of A on C decreases significantly when B is added simultaneously with A as a predictor of C. According to Matthews et al. (2018), if the indirect effect is significant while the direct insignificant, full mediation has occurred, while if both direct and indirect effects are substantial, partial mediation has occurred. This study evidenced that there is partial mediation in the proposed construct (Table 5). Following Preacher and Hayes (2008) this study evidenced that there is partial mediation in the proposed construct, because the relationship between independent variable (social media use) and dependent variable (psychological well-being) is significant (*p*-value < 0.05) and indirect effect among them after introducing mediator (bonding social capital, bridging social capital, social isolation, and smartphone addiction) is also significant (*p*-value < 0.05), therefore it is evidenced that when there is a significant effect both direct and indirect it's called partial mediation.

## Discussion

The present study reveals that the social and psychological impacts of social media use among University students is becoming more complex as there is continuing advancement in technology, offering a range of affordable interaction opportunities. Based on the 940 valid responses collected, all the hypotheses were accepted (*p* < 0.05).

*H1a* finding suggests that social media use is a significant influencing factor of bonding social capital. This implies that, during a pandemic, social media use enables students to continue their close relationships with family members, friends, and those with whom they have close ties. This finding is in line with prior work of Chan (2015) and Ellison et al. (2007), who evidenced that social bonding capital is predicted by Facebook use and having a mobile phone. *H1b* findings suggest that, when individuals believe that social communication can help overcome obstacles to interaction and encourage more virtual self-disclosure, social media use can improve trust and promote the establishment of social associations, thereby enhancing well-being. These findings are in line with those of Gong et al. (2021), who also witnessed the significant effect of bonding social capital on immigrants' psychological well-being, subsequently calling for the further evidence to confirm the proposed relationship.

The findings of the present study related to *H2a* suggest that students are more likely to use social media platforms to receive more emotional support, increase their ability to mobilize others, and to build social networks, which leads to social belongingness. Furthermore, the findings suggest that social media platforms enable students to accumulate and maintain bridging social capital; further, online classes can benefit students who feel shy when participating in offline classes. This study supports the previous findings of Chan (2015) and Karikari et al. (2017). Notably, the present study is not limited to a single social networking platform, taking instead a holistic view of social media. The *H2b* findings are consistent with those of Bano et al. (2019), who also confirmed the link between bonding social capital and psychological well-being among University students using WhatsApp as social media platform, as well as those of Chen and Li (2017).

The *H3a* findings suggest that, during the COVID-19 pandemic when most people around the world have had limited offline or face-to-face interaction and have used social media to connect with families, friends, and social communities, they have often been unable to connect with them. This is due to many individuals avoiding using social media because of fake news, financial constraints, and a lack of trust in social media; thus, the lack both of offline and online interaction, coupled with negative experiences on social media use, enhances the level of social isolation (Hajek and König, 2021). These findings are consistent with those of Adnan and Anwar (2020). The *H3b* suggests that higher levels of social isolation have a negative impact on psychological well-being. These result indicating that, consistent with Choi and Noh (2019), social isolation is negatively and significantly related to psychological well-being.

The *H4a* results suggests that substantial use of social media use leads to an increase in smartphone addiction. These findings are in line with those of Jeong et al. (2016), who stated that the excessive use of smartphones for social media, entertainment (watching videos, listening to music), and playing e-games was more likely to lead to smartphone addiction. These findings also confirm the previous work of Jeong et al. (2016), Salehan and Negahban (2013), and Swar and Hameed (2017). The *H4b* results revealed that a single unit increase in smartphone addiction results in a 6.8% decrease in psychological well-being. These findings are in line with those of Tangmunkongvorakul et al. (2019), who showed that students with higher levels of smartphone addiction had lower psychological well-being scores. These findings also support those of Shoukat (2019), who showed that smartphone addiction inversely influences individuals' mental health.

This suggests that the greater the smartphone addiction, the greater the phubbing. The *H5* findings are in line with those of Chatterjee (2020), Chotpitayasunondh and Douglas (2016), Guazzini et al. (2019), and Tonacci et al. (2019), who also evidenced a significant impact of smartphone addiction and phubbing. Similarly, Chotpitayasunondh and Douglas (2018) corroborated that smartphone addiction is the main predictor of phubbing behavior. However, these findings are inconsistent with those of Vallespín et al. (2017), who found a negative influence of phubbing.

The H6 results suggests that phubbing is one of the significant predictors of psychological well-being. Furthermore, these findings suggest that, when phubbers use a cellphone during interaction with someone, especially during the current pandemic, and they are connected with many family members, friends, and relatives; therefore, this kind of action gives them more satisfaction, which simultaneously results in increased relaxation and decreased depression (Chotpitayasunondh and Douglas, 2018). These findings support those of Davey et al. (2018), who evidenced that phubbing has a significant influence on adolescents and social health students in India.

The findings showed a significant and positive effect of social media use on psychological well-being both through bridging and bonding social capital. However, a significant and negative effect of social media use on psychological well-being through smartphone addiction and through social isolation was also found. Hence, this study provides evidence that could shed light on the contradictory contributions in the literature suggesting both positive (e.g., Chen and Li, 2017; Twenge and Campbell, 2019; Roberts and David, 2020) and negative (e.g., Chotpitayasunondh and Douglas, 2016; Jiao et al., 2017; Choi and Noh, 2019; Chatterjee, 2020) effects of social media use on psychological well-being. This study concludes that the overall impact is positive, despite some degree of negative indirect impact.

### Theoretical Contributions

This study's findings contribute to the current literature, both by providing empirical evidence for the relationships suggested by extant literature and by demonstrating the relevance of adopting a more complex approach that considers, in particular, the indirect effect of social media on psychological well-being. As such, this study constitutes a basis for future research (Van Den Eijnden et al., 2016; Whaite et al., 2018) aiming to understand the impacts of social media use and to find ways to reduce its possible negative impacts.

In line with Kim and Kim (2017), who stressed the importance of heterogeneous social networks in improving social capital, this paper suggests that, to positively impact psychological well-being, social media usage should be associated both with strong and weak ties, as both are important in building social capital, and hence associated with its bonding and bridging facets. Interestingly, though, bridging capital was shown as having the greatest impact on psychological well-being. Thus, the importance of wider social horizons, the inclusion in different groups, and establishing new connections (Putnam, 1995, 2000) with heterogeneous weak ties (Li and Chen, 2014) are highlighted in this paper.

### Practical Contributions

These findings are significant for practitioners, particularly those interested in dealing with the possible negative impacts of social media use on psychological well-being. Although social media use is associated with factors that negatively impact psychological well-being, particularly smartphone addiction and social isolation, these negative impacts can be lessened if the connections with both strong and weak ties are facilitated and featured by social media. Indeed, social media platforms offer several features, from facilitating communication with family, friends, and acquaintances, to identifying and offering access to other people with shared interests. However, it is important to access heterogeneous weak ties (Li and Chen, 2014) so that social media offers access to wider sources of information and new resources, hence enhancing bridging social capital.

### Limitations and Directions for Future Studies

This study is not without limitations. For example, this study used a convenience sampling approach to reach to a large number of respondents. Further, this study was conducted in Mexico only, limiting the generalizability of the results; future research should therefore use a cross-cultural approach to investigate the impacts of social media use on psychological well-being and the mediating role of proposed constructs (e.g., bonding and bridging social capital, social isolation, and smartphone addiction). The sample distribution may also be regarded as a limitation of the study because respondents were mainly well-educated and female. Moreover, although Internet channels represent a particularly suitable way to approach social media users, the fact that this study adopted an online survey does not guarantee a representative sample of the population. Hence, extrapolating the results requires caution, and study replication is recommended, particularly with social media users from other countries and cultures. The present study was conducted in the context of mainly University students, primarily well-educated females, via an online survey on in Mexico; therefore, the findings represent a snapshot at a particular time. Notably, however, the effect of social media use is increasing due to COVID-19 around the globe and is volatile over time.

Two of the proposed hypotheses of this study, namely the expected negative impacts of social media use on social isolation and of phubbing on psychological well-being, should be further explored. One possible approach is to consider the type of connections (i.e., weak and strong ties) to explain further the impact of social media usage on social isolation. Apparently, the prevalence of weak ties, although facilitating bridging social capital, may have an adverse impact in terms of social isolation. Regarding phubbing, the fact that the findings point to a possible positive impact on psychological well-being should be carefully addressed, specifically by psychology theorists and scholars, in order to identify factors that may help further understand this phenomenon. Other suggestions for future research include using mixed-method approaches, as qualitative studies could help further validate the results and provide complementary perspectives on the relationships between the considered variables.

## Data Availability Statement

The raw data supporting the conclusions of this article will be made available by the authors, without undue reservation.

## Ethics Statement

The studies involving human participants were reviewed and approved by Jiangsu University. The patients/participants provided their written informed consent to participate in this study.

## Author Contributions

All authors listed have made a substantial, direct and intellectual contribution to the work, and approved it for publication.

## Conflict of Interest

The authors declare that the research was conducted in the absence of any commercial or financial relationships that could be construed as a potential conflict of interest.

## References

[B1] Abbas R. Mesch G. (2018). Do rich teens get richer? Facebook use and the link between offline and online social capital among Palestinian youth in Israel. Inf. Commun. Soc. 21, 63–79. 10.1080/1369118X.2016.1261168

[B2] Adnan M. Anwar K. (2020). Online learning amid the COVID-19 pandemic: students' perspectives. J. Pedagog. Sociol. Psychol. 2, 45–51. 10.33902/JPSP.202026130933083098

[B3] Ali Qalati S. Li W. Ahmed N. Ali Mirani M. Khan A. (2021). Examining the factors affecting SME performance: the mediating role of social media adoption. Sustainability 13:75. 10.3390/su13010075

[B4] Bagozzi R. P. Yi Y. (1988). On the evaluation of structural equation models. J. Acad. Mark. Sci. 16, 74–94. 10.1007/BF02723327

[B5] Bagozzi R. P. Yi Y. Phillips L. W. (1991). Assessing construct validity in organizational research. Admin. Sci. Q. 36, 421–458. 10.2307/2393203

[B6] Bano S. Cisheng W. Khan A. N. Khan N. A. (2019). WhatsApp use and student's psychological well-being: role of social capital and social integration. Child. Youth Serv. Rev. 103, 200–208. 10.1016/j.childyouth.2019.06.002

[B7] Barbosa B. Chkoniya V. Simoes D. Filipe S. Santos C. A. (2020). Always connected: generation Y smartphone use and social capital. Rev. Ibérica Sist. Tecnol. Inf. E 35, 152–166.

[B8] Bekalu M. A. McCloud R. F. Viswanath K. (2019). Association of social media use with social well-being, positive mental health, and self-rated health: disentangling routine use from emotional connection to use. Health Educ. Behav. 46(2 Suppl), 69S−80S. 10.1177/1090198119863768 31742462

[B9] Brown G. Michinov N. (2019). Measuring latent ties on Facebook: a novel approach to studying their prevalence and relationship with bridging social capital. Technol. Soc. 59:101176. 10.1016/j.techsoc.2019.101176

[B10] Campbell D. T. Fiske D. W. (1959). Convergent and discriminant validation by the multitrait-multimethod matrix. Psychol. Bull. 56, 81–105. 10.1037/h0046016 13634291

[B11] Carlson J. R. Zivnuska S. Harris R. B. Harris K. J. Carlson D. S. (2016). Social media use in the workplace: a study of dual effects. J. Org. End User Comput. 28, 15–31. 10.4018/JOEUC.2016010102

[B12] Chan M. (2015). Mobile phones and the good life: examining the relationships among mobile use, social capital and subjective well-being. New Media Soc. 17, 96–113. 10.1177/1461444813516836

[B13] Chappell N. L. Badger M. (1989). Social isolation and well-being. J. Gerontol. 44, S169–S176. 10.1093/geronj/44.5.s169 2768776

[B14] Chatterjee S. (2020). Antecedents of phubbing: from technological and psychological perspectives. J. Syst. Inf. Technol. 22, 161–118. 10.1108/JSIT-05-2019-0089

[B15] Chen H.-T. Li X. (2017). The contribution of mobile social media to social capital and psychological well-being: examining the role of communicative use, friending and self-disclosure. Comput. Hum. Behav. 75, 958–965. 10.1016/j.chb.2017.06.011

[B16] Choi D.-H. Noh G.-Y. (2019). The influence of social media use on attitude toward suicide through psychological well-being, social isolation, and social support. Inf. Commun. Soc. 23, 1–17. 10.1080/1369118X.2019.1574860

[B17] Chotpitayasunondh V. Douglas K. M. (2016). How phubbing becomes the norm: the antecedents and consequences of snubbing via smartphone. Comput. Hum. Behav. 63, 9–18. 10.1016/j.chb.2016.05.018

[B18] Chotpitayasunondh V. Douglas K. M. (2018). The effects of phubbing on social interaction. J. Appl. Soc. Psychol. 48, 304–316. 10.1111/jasp.12506

[B19] Cohen J. (1998). Statistical Power Analysis for the Behavioural Sciences. Hillsdale, NJ: Lawrence Erlbaum Associates.

[B20] Davey S. Davey A. Raghav S. K. Singh J. V. Singh N. Blachnio A.. (2018). Predictors and consequences of phubbing among adolescents and youth in India: an impact evaluation study. J. Fam. Community Med. 25, 35–42. 10.4103/jfcm.JFCM_71_17 29386960 PMC5774041

[B21] David M. E. Roberts J. A. Christenson B. (2018). Too much of a good thing: investigating the association between actual smartphone use and individual well-being. Int. J. Hum. Comput. Interact. 34, 265–275. 10.1080/10447318.2017.1349250

[B22] Dhir A. Yossatorn Y. Kaur P. Chen S. (2018). Online social media fatigue and psychological wellbeing—a study of compulsive use, fear of missing out, fatigue, anxiety and depression. Int. J. Inf. Manag. 40, 141–152. 10.1016/j.ijinfomgt.2018.01.012

[B23] Dutot V. Bergeron F. (2016). From strategic orientation to social media orientation: improving SMEs' performance on social media. J. Small Bus. Enterp. Dev. 23, 1165–1190. 10.1108/JSBED-11-2015-0160

[B24] Ellison N. B. Steinfield C. Lampe C. (2007). The benefits of Facebook friends: Social capital and college students' use of online social network sites. J. Comput. Mediat. Commun. 12, 1143–1168. 10.1111/j.1083-6101.2007.00367.x

[B25] Fan M. Huang Y. Qalati S. A. Shah S. M. M. Ostic D. Pu Z. (2021). Effects of information overload, communication overload, and inequality on digital distrust: a cyber-violence behavior mechanism. Front. Psychol. 12:643981. 10.3389/fpsyg.2021.643981 33959073 PMC8093436

[B26] Fornell C. Larcker D. F. (1981). Evaluating structural equation models with unobservable variables and measurement error. J. Market. Res. 18, 39–50. 10.1177/002224378101800104

[B27] Gökçearslan S. Uluyol Ç. Sahin S. (2018). Smartphone addiction, cyberloafing, stress and social support among University students: a path analysis. Child. Youth Serv. Rev. 91, 47–54. 10.1016/j.childyouth.2018.05.036

[B28] Gong S. Xu P. Wang S. (2021). Social capital and psychological well-being of Chinese immigrants in Japan. Int. J. Environ. Res. Public Health 18:547. 10.3390/ijerph18020547 33440737 PMC7826720

[B29] Guazzini A. Duradoni M. Capelli A. Meringolo P. (2019). An explorative model to assess individuals' phubbing risk. Fut. Internet 11:21. 10.3390/fi11010021

[B30] Hair J. F. Risher J. J. Sarstedt M. Ringle C. M. (2019). When to use and how to report the results of PLS-SEM. Eur. Bus. Rev. 31, 2–24. 10.1108/EBR-11-2018-0203

[B31] Hair J. F. Sarstedt M. Pieper T. M. Ringle C. M. (2012). The use of partial least squares structural equation modeling in strategic management research: a review of past practices and recommendations for future applications. Long Range Plann. 45, 320–340. 10.1016/j.lrp.2012.09.008

[B32] Hair J. F. Sarstedt M. Ringle C. M. Gudergan S. P. (2017). Advanced Issues in Partial Least Squares Structural Equation Modeling. Thousand Oaks, CA: Sage.

[B33] Hajek A. König H.-H. (2021). Social isolation and loneliness of older adults in times of the CoViD-19 pandemic: can use of online social media sites and video chats assist in mitigating social isolation and loneliness? Gerontology 67, 121–123. 10.1159/000512793 33264778 PMC7801987

[B34] Henseler J. Ringle C. M. Sinkovics R. R. (2009). The use of partial least squares path modeling in international marketing, in New Challenges to International Marketing, Vol. 20, eds R.R. Sinkovics and P.N. Ghauri (Bigley: Emerald), 277–319.

[B35] Holliman A. J. Waldeck D. Jay B. Murphy S. Atkinson E. Collie R. J.. (2021). Adaptability and social support: examining links with psychological wellbeing among UK students and non-students. Fron. Psychol. 12:636520. 10.3389/fpsyg.2021.636520 33613406 PMC7894575

[B36] Jeong S.-H. Kim H. Yum J.-Y. Hwang Y. (2016). What type of content are smartphone users addicted to? SNS vs. games. Comput. Hum. Behav. 54, 10–17. 10.1016/j.chb.2015.07.035

[B37] Jiao Y. Jo M.-S. Sarigöllü E. (2017). Social value and content value in social media: two paths to psychological well-being. J. Org. Comput. Electr. Commer. 27, 3–24. 10.1080/10919392.2016.1264762

[B38] Jordan P. J. Troth A. C. (2019). Common method bias in applied settings: the dilemma of researching in organizations. Austr. J. Manag. 45, 3–14. 10.1177/0312896219871976

[B39] Karikari S. Osei-Frimpong K. Owusu-Frimpong N. (2017). Evaluating individual level antecedents and consequences of social media use in Ghana. Technol. Forecast. Soc. Change 123, 68–79. 10.1016/j.techfore.2017.06.023

[B40] Kemp S. (January 30, 2020). Digital 2020: 3.8 billion people use social media. *We Are Social*. Available online at: https://wearesocial.com/blog/2020/01/digital-2020-3-8-billion-people-use-social-media.

[B41] Kim B. Kim Y. (2017). College students' social media use and communication network heterogeneity: implications for social capital and subjective well-being. Comput. Hum. Behav. 73, 620–628. 10.1016/j.chb.2017.03.033

[B42] Kim K. Milne G. R. Bahl S. (2018). Smart phone addiction and mindfulness: an intergenerational comparison. Int. J. Pharmaceut. Healthcare Market. 12, 25–43. 10.1108/IJPHM-08-2016-0044

[B43] Kircaburun K. Alhabash S. Tosuntaş S. B. Griffiths M. D. (2020). Uses and gratifications of problematic social media use among University students: a simultaneous examination of the big five of personality traits, social media platforms, and social media use motives. Int. J. Mental Health Addict. 18, 525–547. 10.1007/s11469-018-9940-6

[B44] Leong L.-Y. Hew T.-S. Ooi K.-B. Lee V.-H. Hew J.-J. (2019). A hybrid SEM-neural network analysis of social media addiction. Expert Syst. Appl. 133, 296–316. 10.1016/j.eswa.2019.05.024

[B45] Li L. Griffiths M. D. Mei S. Niu Z. (2020a). Fear of missing out and smartphone addiction mediates the relationship between positive and negative affect and sleep quality among Chinese University students. Front. Psychiatr. 11:877. 10.3389/fpsyt.2020.00877 33192635 PMC7481466

[B46] Li W. Qalati S. A. Khan M. A. S. Kwabena G. Y. Erusalkina D. Anwar F. (2020b). Value co-creation and growth of social enterprises in developing countries: moderating role of environmental dynamics. Entrep. Res. J. 2020:20190359. 10.1515/erj-2019-0359

[B47] Li X. Chen W. (2014). Facebook or Renren? A comparative study of social networking site use and social capital among Chinese international students in the United States. Comput. Hum. Behav. 35, 116–123. 10.1016/j.chb.2014.02.012

[B48] Matthews L. Hair J. F. Matthews R. (2018). PLS-SEM: the holy grail for advanced analysis. Mark. Manag. J. 28, 1–13.

[B49] Meshi D. Cotten S. R. Bender A. R. (2020). Problematic social media use and perceived social isolation in older adults: a cross-sectional study. Gerontology 66, 160–168. 10.1159/000502577 31522178

[B50] Mou J. Shin D.-H. Cohen J. (2017). Understanding trust and perceived usefulness in the consumer acceptance of an e-service: a longitudinal investigation. Behav. Inf. Technol. 36, 125–139. 10.1080/0144929X.2016.1203024

[B51] Nunnally J. (1978). Psychometric Methods. New York, NY: McGraw-Hill.

[B52] Oghazi P. Karlsson S. Hellström D. Hjort K. (2018). Online purchase return policy leniency and purchase decision: mediating role of consumer trust. J. Retail. Consumer Serv. 41, 190–200.

[B53] Pang H. (2018). Exploring the beneficial effects of social networking site use on Chinese students' perceptions of social capital and psychological well-being in Germany. Int. J. Intercult. Relat. 67, 1–11. 10.1016/j.ijintrel.2018.08.002

[B54] Podsakoff P. M. MacKenzie S. B. Lee J.-Y. Podsakoff N. P. (2003). Common method biases in behavioral research: a critical review of the literature and recommended remedies. J. Appl. Psychol. 88, 879–903. 10.1037/0021-9010.88.5.879 14516251

[B55] Podsakoff P. M. Organ D. W. (1986). Self-reports in organizational research: problems and prospects. J. Manag. 12, 531–544. 10.1177/0149206386012004088452065

[B56] Preacher K. J. Hayes A. F. (2008). Asymptotic and resampling strategies for assessing and comparing indirect effects in multiple mediator models. Behav Res. Methods 40, 879–891. 10.3758/brm.40.3.879 18697684

[B57] Primack B. A. Shensa A. Sidani J. E. Whaite E. O. yi Lin L. Rosen D.. (2017). Social media use and perceived social isolation among young adults in the US. Am. J. Prev. Med. 53, 1–8. 10.1016/j.amepre.2017.01.010 28279545 PMC5722463

[B58] Putnam R. D. (1995). Tuning in, tuning out: the strange disappearance of social capital in America. Polit. Sci. Polit. 28, 664–684. 10.2307/420517

[B59] Putnam R. D. (2000). Bowling Alone: The Collapse and Revival of American Community. New York, NY: Simon and Schuster.

[B60] Qalati S. A. Ostic D. Fan M. Dakhan S. A. Vela E. G. Zufar Z.. (2021). The general public knowledge, attitude, and practices regarding COVID-19 during the lockdown in Asian developing countries. Int. Q. Commun. Health Educ. 2021:272684X211004945. 10.1177/0272684X211004945 33832371 PMC8058537

[B61] Reer F. Tang W. Y. Quandt T. (2019). Psychosocial well-being and social media engagement: the mediating roles of social comparison orientation and fear of missing out. New Media Soc. 21, 1486–1505. 10.1177/1461444818823719

[B62] Ringle C. Wende S. Becker J. (2015). SmartPLS 3 [software]. Bönningstedt: SmartPLS.

[B63] Ringle C. M. Sarstedt M. Straub D. (2012). A critical look at the use of PLS-SEM in MIS Quarterly. *MIS Q*. 36, iii–xiv. 10.2307/41410402

[B64] Roberts J. A. David M. E. (2020). The social media party: fear of missing out (FoMO), social media intensity, connection, and well-being. Int. J. Hum. Comput. Interact. 36, 386–392. 10.1080/10447318.2019.1646517

[B65] Salehan M. Negahban A. (2013). Social networking on smartphones: when mobile phones become addictive. Comput. Hum. Behav. 29, 2632–2639. 10.1016/j.chb.2013.07.003

[B66] Sarstedt M. Cheah J.-H. (2019). Partial least squares structural equation modeling using SmartPLS: a software review. J. Mark. Anal. 7, 196–202. 10.1057/s41270-019-00058-3

[B67] Schinka K. C. VanDulmen M. H. Bossarte R. Swahn M. (2012). Association between loneliness and suicidality during middle childhood and adolescence: longitudinal effects and the role of demographic characteristics. J. Psychol. Interdiscipl. Appl. 146, 105–118. 10.1080/00223980.2011.584084 22303615

[B68] Shi S. Mu R. Lin L. Chen Y. Kou G. Chen X.-J. (2018). The impact of perceived online service quality on swift guanxi. Internet Res. 28, 432–455. 10.1108/IntR-12-2016-0389

[B69] Shoukat S. (2019). Cell phone addiction and psychological and physiological health in adolescents. EXCLI J. 18, 47–50. 10.17179/excli2018-2006 30956638 PMC6449671

[B70] Shrestha N. (2021). Factor analysis as a tool for survey analysis. Am. J. Appl. Math. Stat. 9, 4–11. 10.12691/ajams-9-1-215330692

[B71] Stouthuysen K. Teunis I. Reusen E. Slabbinck H. (2018). Initial trust and intentions to buy: The effect of vendor-specific guarantees, customer reviews and the role of online shopping experience. Electr. Commer. Res. Appl. 27, 23–38. 10.1016/j.elerap.2017.11.002

[B72] Swar B. Hameed T. (2017). Fear of missing out, social media engagement, smartphone addiction and distraction: moderating role of self-help mobile apps-based interventions in the youth*, Paper presented at the 10th International Conference on Health Informatics* (Porto).

[B73] Tangmunkongvorakul A. Musumari P. M. Thongpibul K. Srithanaviboonchai K. Techasrivichien T. Suguimoto S. P.. (2019). Association of excessive smartphone use with psychological well-being among University students in Chiang Mai, Thailand. PLoS ONE 14:e0210294. 10.1371/journal.pone.0210294 30615675 PMC6322718

[B74] Tateno M. Teo A. R. Ukai W. Kanazawa J. Katsuki R. Kubo H.. (2019). Internet addiction, smartphone addiction, and hikikomori trait in Japanese young adult: social isolation and social network. Front. Psychiatry 10:455. 10.3389/fpsyt.2019.00455 31354537 PMC6635695

[B75] Tefertiller A. C. Maxwell L. C. Morris D. L. (2020). Social media goes to the movies: fear of missing out, social capital, and social motivations of cinema attendance. Mass Commun. Soc. 23, 378–399. 10.1080/15205436.2019.1653468

[B76] Tehseen S. Qureshi Z. H. Johara F. Ramayah T. (2020). Assessing dimensions of entrepreneurial competencies: a type II (reflective-formative) measurement approach using PLS-SEM. J. Sustain. Sci. Manage. 15, 108–145.

[B77] Tehseen S. Ramayah T. Sajilan S. (2017). Testing and controlling for common method variance: a review of available methods. J. Manag. Sci. 4, 146–165. 10.20547/jms.2014.1704202

[B78] Tonacci A. Billeci L. Sansone F. Masci A. Pala A. P. Domenici C.. (2019). An innovative, unobtrusive approach to investigate smartphone interaction in nonaddicted subjects based on wearable sensors: a pilot study. Medicina (Kaunas) 55:37. 10.3390/medicina55020037 30720738 PMC6409719

[B79] Twenge J. M. Campbell W. K. (2019). Media use is linked to lower psychological well-being: evidence from three datasets. Psychiatr. Q. 90, 311–331. 10.1007/s11126-019-09630-7 30859387

[B80] Vallespín M. Molinillo S. Muñoz-Leiva F. (2017). Segmentation and explanation of smartphone use for travel planning based on socio-demographic and behavioral variables. Ind. Manag. Data Syst. 117, 605–619. 10.1108/IMDS-03-2016-0089

[B81] Van Den Eijnden R. J. Lemmens J. S. Valkenburg P. M. (2016). The social media disorder scale. Comput. Hum. Behav. 61, 478–487. 10.1016/j.chb.2016.03.038

[B82] Whaite E. O. Shensa A. Sidani J. E. Colditz J. B. Primack B. A. (2018). Social media use, personality characteristics, and social isolation among young adults in the United States. Pers. Indiv. Differ. 124, 45–50. 10.1016/j.paid.2017.10.030 PMC13309234 42367331

[B83] Williams D. (2006). On and off the'net: scales for social capital in an online era. J. Comput. Mediat. Commun. 11, 593–628. 10.1016/j.1083-6101.2006.00029.x

